# Probing Ultrafast Dynamics of Ferroelectrics by Time‐Resolved Pump‐Probe Spectroscopy

**DOI:** 10.1002/advs.202102488

**Published:** 2021-10-10

**Authors:** Yuan Zhang, Junfeng Dai, Xiangli Zhong, Dongwen Zhang, Gaokuo Zhong, Jiangyu Li

**Affiliations:** ^1^ Shenzhen Key Laboratory of Nanobiomechanics Shenzhen Institute of Advanced Technology Chinese Academy of Sciences Shenzhen Guangdong 518055 China; ^2^ Shenzhen Institute for Quantum Science and Engineering Southern University of Science and Technology Shenzhen Guangdong 518055 China; ^3^ School of Materials Science and Engineering Xiangtan University Xiangtan Hunan 411105 China; ^4^ Department of Physics College of Liberal Arts and Sciences National University of Defense Technology Changsha Hunan 410073 China; ^5^ Department of Materials Science and Engineering Southern University of Science and Technology Shenzhen Guangdong 518055 China; ^6^ Guangdong Provincial Key Laboratory of Functional Oxide Materials and Devices Southern University of Science and Technology Shenzhen Guangdong 518055 China

**Keywords:** ferroeletrics, time‐resolved pump‐probe spectroscopy, ultrafast dynamics

## Abstract

Ferroelectric materials have been a key research topic owing to their wide variety of modern electronic and photonic applications. For the quick exploration of higher operating speed, smaller size, and superior efficiencies of novel ferroelectric devices, the ultrafast dynamics of ferroelectrics that directly reflect their respond time and lifetimes have drawn considerable attention. Driven by time‐resolved pump‐probe spectroscopy that allows for probing, controlling, and modulating dynamic processes of ferroelectrics in real‐time, much research efforts have been made to understand and exploit the ultrafast dynamics of ferroelectric. Herein, the current state of ultrafast dynamic features of ferroelectrics tracked by time‐resolved pump‐probe spectroscopy is reviewed, which includes ferroelectrics order parameters of polarization, lattice, spin, electronic excitation, and their coupling. Several potential perspectives and possible further applications combining ultrafast pump‐probe spectroscopy and ferroelectrics are also presented. This review offers a clear guidance of ultrafast dynamics of ferroelectric orders, which may promote the rapid development of next‐generation devices.

## Graphical abstract

In this review, the ultrafast dynamic features of ferroelectrics, including polarization, lattice, spin, electronic excitation, and their coupling, tracing by time‐resolved pump‐probe spectroscopy are systematically discussed and summarized. The various potential perspectives on ultrafast pump‐probe spectroscopy and the additional nondestructive functionality for ultrafast ferroelectric device design are also presented.

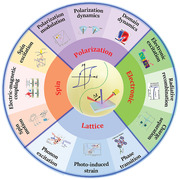

## 1 Introduction

**Figure 1:**
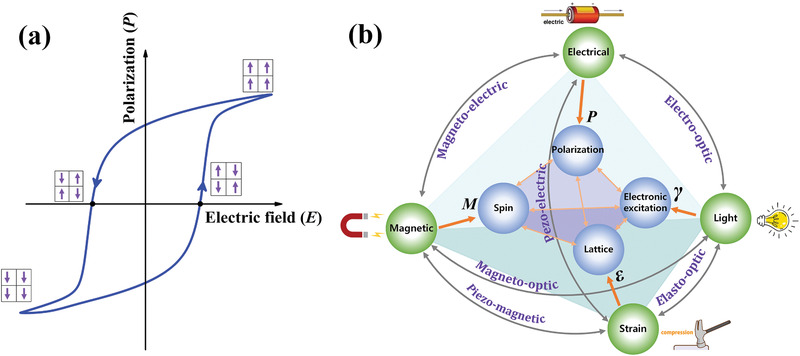
a) Polarization versus electric field loop in ferroelectrics. b) Schematic diagram of multi‐field coupling between polarization, lattice, spin, and photo‐induced electronic excitation in ferroelectrics. The polarization (*P*), strain (*ε*), magnetization (*M*), and photon (*γ*) are controlled by external field of electrical, strain, magnetic, and light, respectively.

Nowadays,microelectronic devices are widely applied in various fields, and the demands for higher transmission speed, multifunctional and artificial intelligent devices are substantially increased.^[^ ^1^, ^2^, ^3^, ^4^ ^]^ With the great desire for high‐speed, miniature, and intelligent functional materials,^[^ ^5^, ^6^ ^]^ the “smart” ferroelectric offering distinct advantages of rapid response speed, non‐volatility, and low power consumption has garnered significant researches.^[^ ^7^, ^8^, ^9^, ^10^, ^11^, ^12^ ^]^ Ferroelectric is a certain type of material that exhibits spontaneous asymmetric crystal structure with permanent electric dipole moments^[^ ^7^, ^13^ ^]^ (**Figure** **1**a), and the spontaneous polarization possesses at least two energetically degenerate crystallographic directions. As the polarization can be coupled with other intrinsic properties as well as external stimuli, the ferroelectric exhibits various physical properties, and is considered as a critical component in modern and future electronic elements.^[^ ^14^, ^15^, ^16^, ^17^, ^18^, ^19^ ^]^ As shown in Figure 1b, under the external stimuli of electric, stress, light, and magnetic fields, the ferroelectric enjoys multiple coupling effects of piezoelectric,^[^ ^20^ ^]^ electric‐optic,^[^ ^21^, ^22^, ^23^ ^]^ magneto‐electric,^[^ ^24^, ^25^ ^]^ piezo‐magnetic,^[^ ^26^ ^]^ and magneto‐optic^[^ ^27^ ^]^ effects, among others.^[^ ^28^, ^29^, ^30^ ^]^ Based on these unusual physical properties, the ferroelectric materials have been employed for widespread applications such as pyroelectric sensors, piezoelectric actuators, electro‐optic modulators, and nonvolatile memories,^[^ ^28^, ^29^, ^30^ ^]^ and other novel applications following the exploration of unusual polarization domain structures have also been conceived.

With the rapid development of ferroelectric materials and devices, the physical mechanism and origin of ferroelectricity are being extensively studied. The multiple coupling effects of ferroelectric are widely demonstrated to be directly associated with the cross‐coupled order parameters of polarization, lattice, spin, and electronic excitation, as shown in Figure 1b.^[^ ^31^, ^32^, ^33^, ^34^, ^35^ ^]^ As the coupled interactions of the aforementioned order parameters possess different origin and efficiencies that lead to various response time, speed, and relaxation processes,^[^ ^31^ ^]^ the time needed for ferroelectric parameter to respond is determined by the ultrafast dynamics of ferroelectrics.^[^ ^36^, ^37^, ^38^, ^39^, ^40^, ^41^ ^]^ For example, the operating speed of ferroelectric memory are dependent on the ultrafast dynamics of polarization switching.^[^ ^42^ ^]^ The function as well as performance of electronic and photonic applications of sensor, IR detector, and waveguide devices rely on the dynamics behaviors between lattice and polarization order parameter of ferroelectrics.^[^ ^20^, ^26^, ^43^ ^]^ The dynamic processes of ferroelectrics not only determine the operating speed of ferroelectric‐based devices, but also decide their lifetimes and efficiencies.^[^ ^42^ ^]^ Meaningfully, when the size of ferroelectric materials and devices is reduced, the dynamic processes of ferroelectric become more significant, since the response speeds of devices are increased with the rapid scaling down of devices.^[^ ^5^, ^6^ ^]^ Apparently, in the pursuit of ferroelectric devices with superior operating speed, efficiencies, and smaller size, a clear understanding of dynamic behavior of ferroelectric are required.

**Figure 2:**
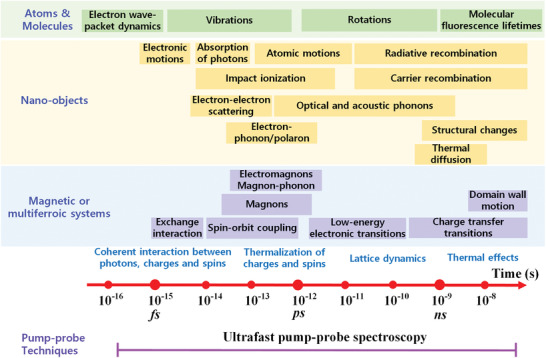
Timescale of ultrafast dynamics in atoms, molecules, nano‐objects, and magnetic or multiferroic systems.

Recently, the dynamical processes of ferroelectric are under significant investigations, and numerous effort have been made to track their ultrafast dynamic behaviors in real‐time.^[^ ^36^, ^37^, ^38^, ^39^, ^40^, ^41^ ^]^ The ultrafast dynamics of polarization reversal, lattice, and spin excitation, photo‐induced electronic excitation, as well as coupled magnetic and ferroelectric order are of considerable interests, which typically occur in femtosecond to nanosecond time scale as schematically presented in **Figure** **2**.^[^ ^39^, ^42^, ^43^ ^]^ As the short electrical pulses are complicated by difficulties to trap the ultrafast dynamics of ferroelectrics, a powerful time‐resolved pump‐probe spectroscopy with higher temporal resolution up to femtoseconds have been widely developed and applied.^[^ ^44^, ^45^, ^46^, ^47^, ^48^, ^49^ ^]^ Amazingly, the ultrafast pump‐probe spectroscopy also provide photo‐physical and structural‐functional properties of ferroelectrics.^[^ ^50^ ^]^ Until now, a greater number of dynamical processes in ferroelectrics probed by time‐resolved pump‐probe spectroscopy have been reported, including polarization dynamics,^[^ ^42^ ^]^ ultrafast polarization modulation,^[^ ^51^ ^]^ light induced/photoassisted polarization,^[^ ^52^ ^]^ photoinduced mechanical strain,^[^ ^53^ ^]^ phase transition,^[^ ^41^ ^]^ electron‐phonon/phonon‐polariton/electric‐magnetic coupling,^[^ ^54^ ^]^ electronic/coherent phonon/lattice vibration excitation,^[^ ^55^, ^56^ ^]^ carrier dynamics and radiative recombination,^[^ ^57^ ^]^ among others. While the ultrafast dynamics of ferroelectrics is an important research topic and have fostered many fascinating studies, there is still lack of a systematic review. Classifying the dynamical process of ferroelectric is becoming essential, for both understand and design the time‐resolved operation of ferroelectric devices.

Herein, the dynamical processes of ferroelectrics probed by time‐resolved pump‐probe spectroscopy are summarized and reviewed. In Section 2, the time‐resolved pump‐probe technique is introduced, and in Section 3 to Section 6 the dynamical processes of polarization, lattice, spin, electronic excitation and their coupling are presented. In Section 7, the applications of pump‐probe spectroscopy in ferroelectrics are summarized, and in Section 8 the prospect of pump‐probe spectroscopy in ferroelectrics are discussed.

## 2 Ultrafast Pump‐Probe Spectroscopy

### 2.1 Fundamentals of Nonlinear Optics

The optical process of materials under optical field can be expressed by expanding the macroscopic polarization *P*, as follows:^[^ ^58^, ^59^ ^]^

(1) $$P=\varepsilon_{0}\left[\chi_{1}+\chi_{2}:E+\chi_{3}\vdots \mathrm{EE}+\cdots \right]E$$

where *ɛ_0_*, *χ*_1_and *χ*_*n*_(*n* ≥ 2) represent the dielectric constant of free space, linear optical susceptibility, and *n*‐th‐order nonlinear susceptibility, respectively. The linear optical susceptibility corresponds to conventional linear optical effects such as refraction and absorption. The typical second‐order nonlinear optical effects include second‐harmonic generation (SHG), sum‐ and difference‐frequency generation, optical rectification and Pockels effect, which can be described by *χ*_2_ susceptibility. The third‐order nonlinear optical effects usually contain third‐harmonic generation, four‐wave mixing and intensity‐dependent refractive index changes, which normally arise from *χ*_3_ susceptibility.^[^ ^58^ ^]^ Besides involving photons that are nonlinear dependence on optical field *E*, the nonlinear optical processes of medium also become nonlinear indirectly through other types of excitations, as follows:^[^ ^58^ ^]^

(2) $$\begin{array}{ccc} P & = & \varepsilon_{0}\left[\chi_{1}+\chi_{2}:E+\chi_{3}\vdots EE+\cdots \right]E\\ & = & \varepsilon_{0}\left[\chi_{q}:Q+\chi_{a}:S+\chi_{\eta }\vdots \eta \ldots \right]E \end{array}$$

As can be found in Equation (2), the optical susceptibility is as a function of molecular vibrational amplitude *Q*, which involve interaction of laser light and molecular vibrational and rotations (in liquids, gases), or optical phonon (in solids) that can lead to stimulated Raman. As the stress are associated with an acoustic wave *S*, the nonlinear optical in the medium can also involve laser and acoustic waves or acoustic phonons that can lead to stimulated Brillouin. In additions, the combined excitations with an amplitude *η* of any space‐charge or plasma wave may lead to a polariton that can be observed during optical nonlinear process, wherein the laser and mixed excitations of photons and phonons can lead to the simulated polariton process. Overall, the ferroelectrics upon optical pulse irradiation would experience several regimes of excitations, which provide different relaxation processes with various time scales before returning to the original equilibrium state.

### 2.2 Fundamentals of Ultrafast Pump‐Probe Spectroscopy

**Figure 3:**
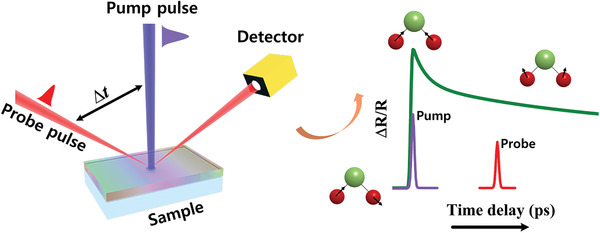
The schematic illustration of the pump‐probe spectroscopy in reflectivity.

In the pump‐probe spectroscopy, the ultrafast pulse train generated from laser can be divided into two kinds, and the time resolution ∆*t* is achieved by introduce the mechanical delay stage that transformed time resolution measurement into space resolution (10 fs in time corresponds to 3 µm in space).^[^ ^44^ ^]^ As the schematic illustration shown in **Figure** **3**, the first pulse train is used to excite sample (called pump pulse), and the second pulse train (called probe pulse) is used to observe the pump‐induced changes.^[^ ^48^ ^]^ The pump excitations in ferroelectrics usually contain ultrafast optical pulse and Terahertz (THz, 1 THz = 10^12^ Hz, photon energy ≈4 meV at 1 THz) waves, wherein the THz waves are electromagnetic waves in the range from 0.3 to 30 THz.^[^ ^60^ ^]^ Under the excitations of ultrafast optical, the structural, electronic, and magnetism of a material would be manipulated,^[^ ^61^, ^62^, ^63^ ^]^ since the created highly non‐thermal electron distribution can be relaxed by electron‐electron scattering and followed by thermalization through coupling with other degrees of freedom.^[^ ^64^ ^]^ Nevertheless, the above‐gap charge excitation induced by visible optical pulse typically results in highly incoherent dynamics with a huge transfer of entropy, which lead to the optical pump with high energy photons (≈eV) presenting a limitation control capability for low‐lying excitations (e.g., vibrational modes of crystal lattice or magnetic excitations).^[^ ^64^ ^]^ For THz waves, it is possible to achieve resonant excitation of vibrational modes with strong transient fields, since the excitation of phonons (local vibrational modes) typically reach with several to dozens THz frequencies and all collective modes of broken symmetry states lie in single‐THz range.^[^ ^60^, ^64^, ^65^ ^]^ The THz waves can drive relevant low‐lying excitations into nonlinear regime and access coherent dynamics with no relevant increase in entropy.^[^ ^64^ ^]^ Indeed, directly access to low‐energy excitations of vibrational modes (e.g., rotational, torsional, phonon), spin waves, and internal excitations of bound electron‐hole pairs by THz waves are achieved.^[^ ^39^, ^66^, ^67^, ^68^, ^69^ ^]^

To investigate the changes of ferroelectrics produced by pump of optical pulse or THz waves, the probing techniques mainly include optical, THz waves, and X‐ray diffraction.^[^ ^39^, ^70^, ^71^, ^72^ ^]^ For optical probing techniques, the common used system is the measurement of transmitted or reflected probe, which can be established into optical pump‐optical probe (OPOP) and THz pump‐optical probe (TPOP) spectroscopy base on the different pump excitation method.^[^ ^57^, ^73^ ^]^ As the SHG process exhibits unique responses to macroscopic polarization, domain structures, and asymmetric lattice structure,^[^ ^74^, ^75^, ^76^, ^77^, ^78^ ^]^ the SHG waves also act as probing pulse in the pump‐probe spectroscopy of ferroelectrics. For the SHG probing techniques, the optical pump‐SHG probe (OPSP)^[^ ^70^ ^]^ and THz pump‐optical probe (TPSP)^[^ ^51^, ^73^ ^]^ spectroscopy are established, and the pump‐induced changes of SHG signals generated from sample are monitored in real‐time. For the THz probe spectroscopy, it is usually established into THz emission spectroscopy (optical pump‐THz probe, OPTP)^[^ ^72^, ^79^ ^]^ or THz time‐domain spectroscopy (THz pump‐THz probe, TPTP),^[^ ^80^, ^81^ ^]^ which is realized by varying the delay between excitation pump pulse (optical or THz) and the THz probe beams. The advantages of THz probing is that it can simultaneous measure the amplitude and phase of THz electric field by exploiting the Pockels effect in electro‐optical crystal, which can obtain the real and imaginary parts of the dielectric response without the need of complex Kramers–Kronig transforms.^[^ ^82^, ^83^ ^]^ For the X‐ray diffractions probing techniques, it can direct track the structural changes of materials, and can be established into optical pump‐X‐ray probe (OPXP)^[^ ^84^ ^]^ and THz pump‐X‐ray probe (TPXP)^[^ ^68^ ^]^ spectroscopy. The X‐ray diffractions probing is achieved with the intensity change of the diffraction peak presented as function of time delay or different pump fields.^[^ ^68^, ^84^ ^]^ In the described ultrafast time‐resolved pump‐probe techniques, the time resolution of OPOP, OPSP, and OPXP spectroscopy can range from femtoseconds to tens of picoseconds or nanoseconds. The time resolution of pump‐probe spectroscopy embodied THz pulses (i.e., OPTP, TPSP, TPTP, TPOP, and TPXP) are always around picosecond, since 1 THz in frequency domain is equal to 1 ps in time domain.^[^ ^85^ ^]^

## 3 Polarization Dynamics of Ferroelectrics

### 3.1 Polarization Dynamics

**Figure 4:**
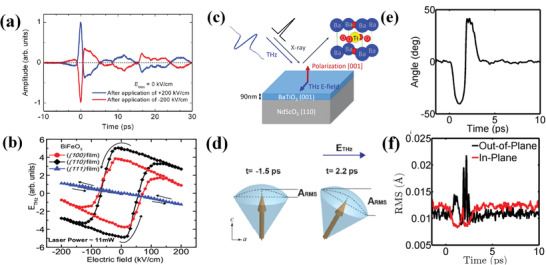
a) Time‐domain waveform of THz pulse radiated from BFO photoconductive switch measure at a zero‐bias electric field, after once applying *E*_bias_ of ±200 kV cm^−1^. The dashed line represented the zero‐level line shown for clarity. Reproduced with permission.^[^ ^72^ ^]^ Copyright 2006, American Physical Society. b) Dependence of *E*_THz_ on the applied *E*_bias_ generates a hysteresis loop for (100)‐ (110)‐ oriented films and an almost linear variation with opposite polarity for (111) films. Reproduced with permission.^[^ ^42^ ^]^ Copyright 2009, Wiley‐VCH. c) Schematic illustration of experimental setup, applied THz field and enhancement electrode structure of BTO sample. d) Schematic illustration of a rotation of polarization leads to an increasing in out‐of‐plane rms displacements. e) The angle of net polarization with respect to *c* axis. f) The rms displacement of Ti atom along in‐plane and out‐of‐plane direction after in‐plane THz excitation. Reproduced with permission.^[^ ^38^ ^]^ Copyright 2016, American Physical Society.

In this section, we would illustrate the ultrafast polarization dynamics of ferroelectrics. As the shape and strength of THz signals generated from ferroelectrics are inextricably linked to the properties of ferroelectric polarization,^[^ ^72^ ^]^ the OPTP spectroscopy has been widely used to study the dynamics of polarization. Taking the typical ferroelectric material BiFeO_3_ (BFO) thin films as examples, under the illumination of femtosecond laser with 400 nm, the photocarriers are excited and the novel THz radiation is observed.^[^ ^72^ ^]^ As shown in **Figure** **4**a, the THz pulse from (001)‐oriented BFO thin films can be radiated at zero bias electric field (*E*_bias_) after once applying initial *E*_bias_ at ± 200 kV cm^−1^. The generated two THz waveforms in BFO thin film exhibit a reversed phase *π*, which are absolutely differed from semiconductor samples that the THz wave is not excited without *E*_bias_ at. It indicates that the THz radiation is directly related to the *P*_s_ state of ferroelectrics, since the poled initial *E*_bias_ would align the spontaneous polarization (*P*_s_) of BFO. These inferences are further confirmed by the given relationship of measured main peak amplitude *E*_THz_ versus *E*_bias_. As shown in Figure 4b, with different initial *E*_bias_ treatment, the *E*_THz_ shows a similar polarization hysteresis loop in (100) and (110) oriented BFO films,^[^ ^42^ ^]^ while the *E*_THz_ versus *E*_bias_ relationship in (111)‐oriented BFO film is linear that is analogous to a nonferroelectric materials. Unlike the (111)‐oriented BFO film that the THZ radiation is resulted from time‐varying current density, the THz emissions in (001)‐and (110)‐oriented BFO films are derived from ultrafast depolarization. It can be found that the ultrafast depolarization of ferroelectric occurs over a time‐scale of 1 to 2 ps. In additions, a series studies of THz emissions from BFO thin films with different thicknesses and structures have been carried out via OPTP spectroscopy.^[^ ^86^, ^87^ ^]^ The results reveal that the THz emission efficiency under zero *E*_bias_ is nearly independent of phase structure, whereas the hysteresis loops of *E*_THz_ versus *E*_bias_ rely on ferroelectric properties, such as lattice structures and leakage current. It can be concluded that the ferroelectric coercivity field exhibits structural lattice independent characteristics, while the ferroelectric polarization switching is lattice dependent.

The dynamics of polarization are also studied by TPXP spectroscopy, since the THz field can be directly used to induce the time‐dependent structural response of ferroelectrics. As shown in Figure 4c, the in‐plane THz field is applied in BaTiO_3_ (BTO) thin film, which is perpendicular to the ferroelectric polarization direction of BTO.^[^ ^38^ ^]^ Upon the THz excitation, as shown in Figure 4d, the dipoles rotate in the transverse electric field of bias pulse, and the intrinsic in‐plane distribution (*t* = −1.5 ps) is transformed to one with an increased component along out‐of‐plane direction (*t* = 2.2 ps). The calculated time‐dependent polarization rotations induced by applied THz field are shown in Figure 4e, which indicates that the rotation amplitudes are ≈40°. The comparison of in‐plane and out‐of‐plane response time dependence rms displacements in Figure 4f show that the in‐plane rms distribution is decreased, whereas the out‐of‐plane distribution is increased. The observed effect is resulted from direct THz‐driven coupling to lattice, which initially drives polar displacements along the direction of applied electric field. Obviously, the THz field can drive large‐amplitude atoms displacements along ferroelectric polarization axis, which are comparable to built‐in displacements associated with intrinsic polarization and incoherent across unit cell.

The ultrafast dynamics of polarization are also observed via OPSP or OPXP spectroscopy.^[^ ^37^, ^51^ ^]^ In the case of LiNbO_3_ (LNO) crystal, the ultrafast optical reversal of ferroelectric polarization has been revealed by OPSP spectroscopy.^[^ ^37^ ^]^ Rather than directly driving the ferroelectric mode, the resonant excitation of an auxiliary high‐frequency phonon mode with femtosecond mid‐IR pulses are indirectly coupled. Owing to the strong anharmonic coupling between these modes, the atoms are directionally displaced along the ferroelectric mode and the polarization is transiently reversed for the time delays between 0 and 0.2 ps. Another case is in the PbZr*_x_* Ti_1−_ *_x_* O_3_/SrRuO_3_ (PZT/SRO) superlattice, the ultrafast dynamics of coupling between lattice and polarization are studied via OPXP spectroscopy.^[^ ^36^ ^]^ The Bragg reflection of two relevant phonon modes of tetragonal distortion and soft mode (0055) and (0056) are chosen to derive in PZT film. The optical excitation of SRO metal layers can generate giant stress (> 1 GPa), which lead to a strong suppression of PZT layers by up to 2%. The maximum changes in tetragonality reaches after 1.3 ps, and the anharmonic coupling of both two modes reduces the ferroelectric polarization up to 100% with a slight delay.

### 3.2 Ultrafast Polarization Modulation

**Figure 5:**
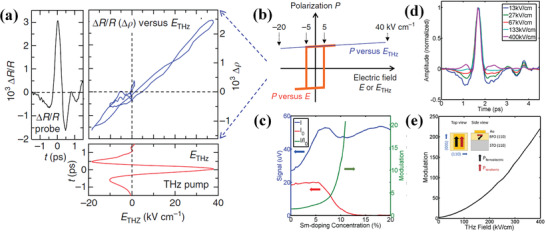
a,b) Changes of polarization *P* induced by THz field *E*_THz_ in TTF‐CA. a) Left panel: Time evolution of reflectivity change *∆R*/*R* induced by *E*_THz_ in ionic phase (78 K) for delay time *t* of −1.5 ps ≤ *t* ≥1.5 ps. Bottom panel: Time evolution of *E*_THz_ for delay time *t*. Right panel: *∆R*/*R* as a function of *E*_THz_ derived from left and bottom figure, where the right vertical axis denotes *∆ρ*. b) Schematics of the *P*–*E* curves for *E*_THz_ (blue line) and for quasi‐static electric field *E* (orange line). a,b) Reproduced with permission.^[^ ^51^ ^]^ Copyright 2013, Nature Publishing Group. c) 100 nm Sm‐BFO film response as a function of Sm‐doping. The static SHG goes to zero, reflecting the centrosymmetry of the paraelectric phase (red curve). The blue curve shows the raw SHG intensity with THz field on. The green curve shows the on‐off ratio. d) Measured normalized electric induced THz signal for different applied THz fields within the electrode structures near the morphotropic phase boundary (≈11% doping). e) The modulation in SHG intensity for Sm‐BFO with electrodes as a function of peak THz field near the morphotropic phase boundary. c‐e) Reproduced with permission.^[^ ^88^ ^]^ Copyright 2015, Wiley‐VCH.

As the timescale of polarization or depolarization movement is demonstrated at picosecond, the THz field is widely used to realize ultrafast control of ferroelectric polarization for their matched picosecond duration.^[^ ^51^, ^88^ ^]^ More importantly, the THz field has a nearly monocyclic electric field even up to 1 MV cm^−1^, which is far larger than the coercive field of polarization reversal.^[^ ^51^ ^]^ The ultrafast modulation of polarization amplitude driven by THz fields is first observed in electronic‐type organic ferroelectrics tetrathiafulvalene‐p‐chloranil (TTF‐CA) via TPOP spectroscopy.^[^ ^51^ ^]^ As shown in **Figure** **5**a, the time evolutions of reflectivity ∆*R*/*R* (left panel) and *E*_THz_(*t*) (bottom panel) within the delay time of −1.5 ps ≤ *t* ≤1.5 ps are comparatively analyzed, to more precisely see the interrelation between ∆*R*/*R*−*E*_THz_(*t*) (right panel) within the duration of a THz pulse. The ∆*R*/*R*−*E*_THz_(*t*) curve can be converted to ∆*ρ*−*E*_THz_(*t*) with *ρ* representing the magnitude of THz induced modulations, and it can be regarded as the ferroelectric polarization *P*–*E*_THz_ curve. The comparison of the obtained *P*–*E*_THz_ curve and the *P*–*E* curve measured by quasi‐static fields (10 Hz at ≈50 K) in Figure 5b reveals that the THz pulse with a high electric field of 40 kV cm^−1^ can lead to a polarization reversal. The sub‐picosecond modulation of ferroelectric polarization amplitude by a THz electric field is enabled. As the domain‐wall motions are much slower than the electric‐field changes within a duration of THz pulse, the produced ultrafast macroscopic polarization in the TTF‐CA is without any domain‐wall motions. Subsequently, the ultrafast polarization modulation are also achieved in inorganic ferroelectrics including BFO and (Ba_0.8_Sr_0.2_)TiO_3_ (BST) thin films via TPSP spectroscopy.^[^ ^88^, ^89^, ^90^ ^]^ Under the action of THz pulse, the ferroelectric order parameter of BST film acquires an in‐plane component up to 6% of the net polarization.^[^ ^89^ ^]^ In the Sm doped BFO films,^[^ ^88^ ^]^ the field‐induced change in SHG intensity and on–off ratio are enhanced near phase boundary (Figure 5c). As shown in Figure 5d,e, the large amplitude modulations of time‐dependent ferroelectric polarization with on–off ratios of 220× gateable are monitored on sub‐picosecond timescales in BFO films. These effects point toward novel applications with respect to ferroelectric photonic switches and electromechanical devices gated by all‐optically applied fields.

Besides rapidly controlling polarizations, the TPOP and TPSP spectroscopy are also proved as powerful tools to clarify the mechanisms of ferroelectricity by probing the transient reflectivity and SHG signals, since the polar ferroelectric can certainly generate SHG signals.^[^ ^78^, ^91^ ^]^ In the case of charge‐order phase of a prototypical ET‐based molecular compound *α*‐(ET)_2_I_3_, after sub‐picosecond polarization modulation by terahertz fields, prominent oscillations only appear in the reflectivity‐probe but not in the SHG‐probe.^[^ ^73^ ^]^ This results suggest that the molecular displacements responsible for the coherent oscillations can stabilize the charge‐order of *α*‐(ET)_2_I_3_, but they are not coupled strongly with ferroelectric polarization. In the case of TTF‐CA, the TPSP spectroscopy is used to detect the sub‐picosecond macroscopic‐polarization generation, and the approach of TPOP spectroscopy is applied to clarify the nature of ferroelectric domain walls.^[^ ^92^ ^]^ A large macroscopic polarization with magnitude reaching to 20% is observed under the THz filed of ≈400 kV cm^−1^, which is explained by the quantum dynamics of neutral‐ionic domain walls pairs via field‐induced intermolecular. The intermolecular charge can transfer and breathe motions of domain walls between microscopic neutral and ionic domains, and the decay time of neutral‐ionic domain walls increases from 0.5 to 3 ps with decreasing temperature.

### 3.3 Domain Dynamics

**Figure 6:**
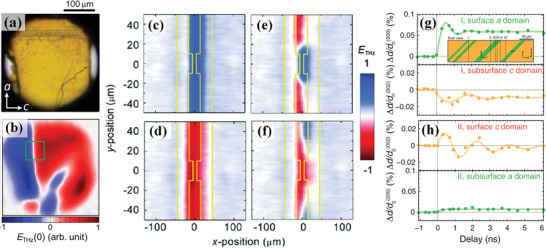
a,b) Visible image on the *ac* plane of ferroeletric croconic acid and the corresponding THz‐radiation image.a,b) Reproduced with permission.^[^ ^93^ ^]^ Copyright 2014, American Institute of Physics. c–f) Visualization of 180° ferroelectric domain structure of the BFO thin film probed with THz amplitude *E*_THz_. The domain structure after poling the BFO′ film with a bias voltage *V*_bias_ of c) +200 V and d) −200 V with simultaneous laser illumination, and domain structure after poling with *V*_bias_ of e) +200 V and f) −200 V without laser illumination. c‐f) Reproduced with permission.^[^ ^72^ ^]^ Copyright 2006, American Physical Society. g,h) The (200) and (002) lattice‐spacing change for *a* and *c* domains from those before *t* = 0, Δ*d*/*d*_0_^(200)^, and Δ*d*/*d*_0_^(002)^, respectively, as a function of time delay at g) *I* and h) *II* locations, where the insert is locations I–VI at which time‐delay dependent XRD measurements performed. g,h) Reproduced with permission.^[^ ^84^ ^]^ Copyright 2018, American Physical Society.

The THz emission via OPTP spectroscopy is also demonstrated as a sensitive tool to probe ferroelectric domain imaging microscopy. By scanning 2D distribution of *E*_THz_, the orientation of 180° domains can be distinguished via signs of *E*_THz_, and the ferroelectric domain structures are visualized.^[^ ^52^, ^72^, ^87^ ^]^ As shown in **Figure** **6**a,b, the ferroelectric domain of organic ferroelectric croconic acid are observed by mapping out the THz radiation.^[^ ^93^ ^]^ It can be seen that the visualized domain sizes are larger than 50 µm square, which are separated by both 180° and tail–tail domain walls. In another case of BFO thin film, four domain images at zero‐bias electric field after poling the film under initial ±200 V bias voltage (*V*_bias_) with and without simultaneous laser illumination are shown in Figure 6c–f.^[^ ^72^ ^]^ As can be seen, domains with opposite polarization states appear as blue and red areas, which are depended on the sign of *E*_THz_. Herein, only the domains between electrodes have changed their state under the applied initial opposite *V*_bias_, whereas the other states remain unchanged and are independent to initial *V*_bias_. The phase of entire domains between the electrodes is managed to reverse *π* by opposite *V*_bias_ (Figure 6c,d) under laser illumination, but only two areas near dipole gap have reversed the domains without laser illumination (Figure 6e,f). These results indicate that the opposite 180° domains are coexistence between the two strip lines, and the two different states with and without laser are derived from “photoassisted *P*_s_ switching”.^[^ ^52^, ^87^ ^]^ As the amplitude of THz radiation is sensitive to the overall sum of electric dipole moments within laser spot area, the illumination of band gap light can create additional switchable ferroelectric dipoles and align more domain area in one direction by depinning domains. The combination of UV light exposure with applied *V*_bias_ shows superior switching ability than the application of *V*_bias_ alone. The significantly larger THz amplitude under laser illumination would be a strong evidence of light‐assisted poling in ferroelectrics.

The dynamics of domain switching are also studied using OPXP spectroscopy. In the case of single crystal BTO, the striking structural and electrical dynamics upon optical excitation can be simultaneously captured on sub‐nanoseconds and nanoscale within an individual ferroelectric domain and across domain walls.^[^ ^84^ ^]^ The BTO crystal contains both *a* and *c* domains, which are separated by 90° domain walls with polarization parallel and normal to the crystal surface, respectively. The surface *a* domain and underlying subsurface *c* domain in BTO sample can be depicted by the lattice spacing of *d* ^(200)^ and *d* ^(002)^. From the time‐delay dependence of (200) and (002) lattice‐spacing intensities in Figure 6g,h, it can be found that the out‐of‐plane lattice spacing increases within the first 100–150 ps under the optical pump pulse. While the surface *a* domain increases and the subsurface *c* domain decreases within the same time period. It indicates that the polarization and lattice dynamics driven by optical are dramatically distinct in a surface layer versus bulk regions, and a large emergent photo‐induced surface layer with electric field up to 20 MV m^−1^ is created after photoexcitation. The temporal electric field would expand *c* domain and shrink *a* domain by activating domain wall motions. The *c* domain presents a subsonic domain growth speed of 2.5 m s^−1^ within the first nanosecond after excitation, and the polarization of *a* domain tilts from the in‐plane to the out‐of‐plane direction by up to 7.5° over 0.5 ns. The advances in spatiotemporal imaging tools of OPXP spectroscopy open opportunities for disentangling ultrafast processes in complex mesoscale structures such as ferroelectric domains.

## 4 Lattice Dynamics of Ferroelectrics

### 4.1 Optical Phonon Excitations (Soft Mode) and Phase Transitions

**Figure 7:**
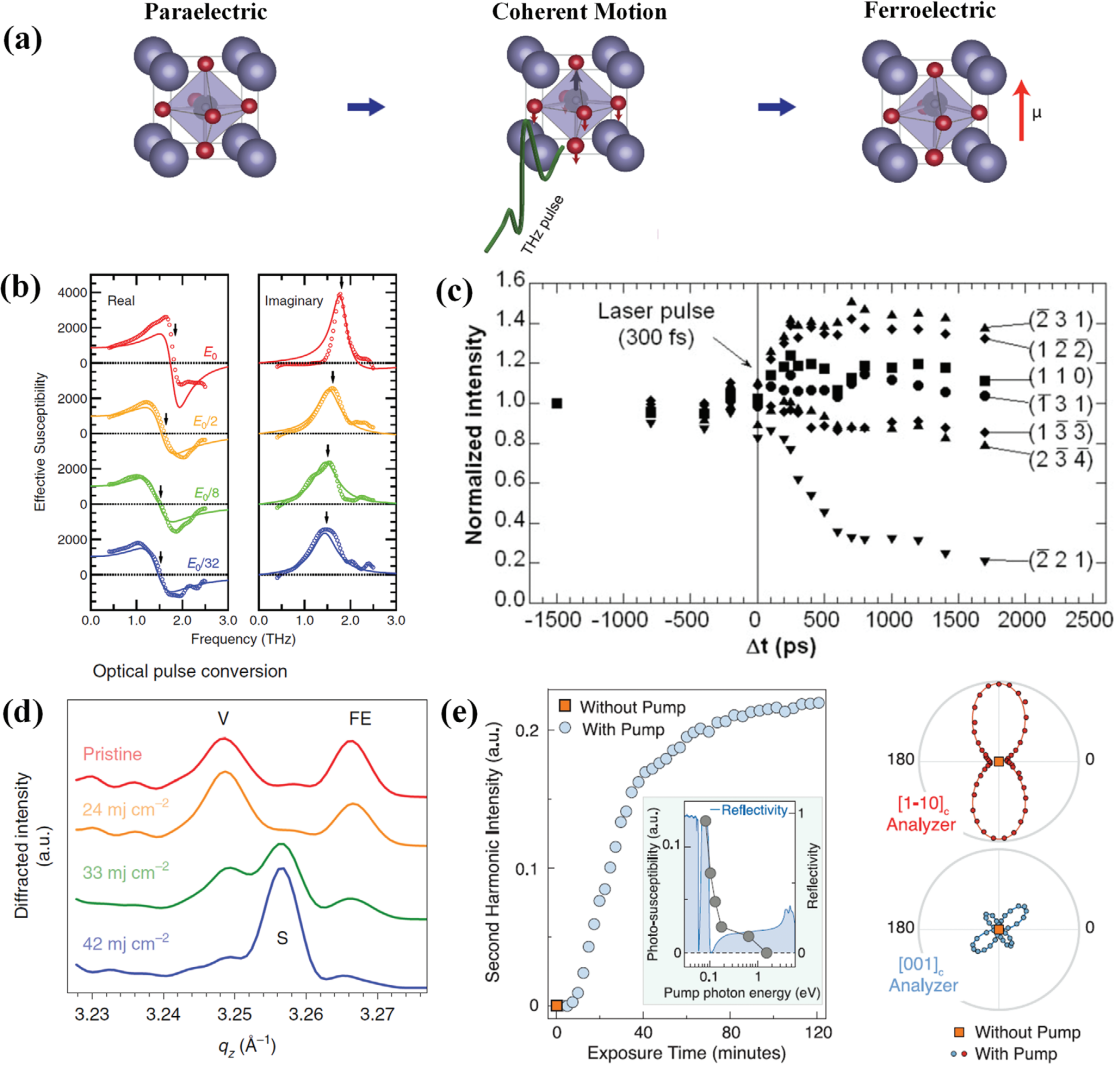
a) A single‐cycle THz frequency electric field moves all the ions it encounters toward their positions in a new crystalline phase in STO. Reproduced with permission.^[^ ^94^ ^]^ Copyright 2019, American Association for the Advancement of Science. b) Derived real and imaginary parts of effective susceptibility *χ*_eff_ ≡ *P*(*ω*)/*E*_film_(*ω*). *E*_0_ is equal to 80 kV cm^−1^. Reproduced with permission.^[^ ^43^ ^]^ Copyright 2012, American Physical Society. c) Relative intensity of some Bragg reflections versus the delay between the laser pump and the X‐ray probe in TTF‐CA. Reproduced with permission.^[^ ^41^ ^]^ Copyright 2003, American Association for the Advancement of Science. d) Superlattice peaks near 002_pc_ (pseudocubic notation) showing two distinct diffraction peaks, corresponding to the V and FE phases in the pristine sample, that transform into a single uniform S phase. Reproduced with permission.^[^ ^96^ ^]^ Copyright 2019, Nature Publishing Group. e) Left panel: Time delay‐independent total SHG intensity impinging on the detector (without analyzer) as a function of exposure time. Right panel: SHG intensity in the saturated state as a function of the probe polarization for two orthogonal analyzer configurations.Reproduced with permission.^[^ ^40^ ^]^ Copyright 2019, American Association for the Advancement of Science.

In ferroelectrics, lattice dynamics are brought into sharp focus, since direct manipulation of atomic lattice using time‐resolved pump‐probe spectroscopy is becoming important. As shown in **Figure** **7**a, under the excitation of THz pulses, the ionic can be impulsively driven to far from thermal equilibrium, and the crystal structure can be dynamically changed.^[^ ^94^ ^]^ As a phonon excitation of soft mode is associated with ferroelectric phase transition, the soft modes are particularly suited to a demonstration of coherent control of terahertz‐induced macroscopic phase transformations.^[^ ^43^ ^]^ Taking SrTiO_3_ (STO) thin films as example, as shown in Figure 7b, the ferroelectric soft mode are impulsively driven to a large amplitude using TPTP spectroscopy, and the soft‐mode absorption peaks exhibit nonlinear response of blueshifting and spectral narrowing with the increasing of THz electric field.^[^ ^43^ ^]^ The induced displacement is comparable to that of the perturbation‐induced ferroelectric phase transition, and these nonlinear responses can be interpreted using a classical anharmonic oscillator model. As the STO has several zone‐center IR active phonon modes within the bandwidth of THz radiation sources that can be driven resonantly,^[^ ^68^ ^]^ the direct structural evidence of phonon up‐conversion has been demonstrated in STO thin film via TPXP spectroscopy.^[^ ^68^ ^]^ The intense single‐cycle terahertz radiation couples directly to the soft mode via IR absorption and drives the system far from equilibrium into the strongly nonlinear regime. Subsequent energy coupling into high‐frequency phonon modes occur at 5.15 and 7.6 THz, which transfer energy to higher‐frequency phonon modes through strongly nonlinear coupling. As the lowest soft phonon modes of ferroelectric are supposed to have a strong influence on the permittivity, the relationship between dielectric properties and polar phonon mode are studied in ferroelectric by TPTP spectroscopy.^[^ ^95^ ^]^ In the case of BFO ceramics, the enhanced permittivity on heating are contributed from the sum of polar phonon, which can be explained by the remarkable lattice softening.^[^ ^82^ ^]^ Regarding to BTO power sample, the low frequency dielectric response are closely related to the lowest pair of transverse optical and longitudinal optical phonon modes, which provides a better understanding of the relation of low‐frequency dielectric function with the optical phonon mode for ferroelectric materials.^[^ ^95^ ^]^

Actually, the phase transition routes in ferroelectrics under the excitation of femtosecond laser or THz pulses contain two different paths of equilibrium or non‐equilibrium.^[^ ^41^ ^]^ In the equilibrium route, the photoinduced paraelectric to ferroelectric structural phase transition has been directly observed in an organic charge‐transfer crystal TTF‐CA via OPXP spectroscopy.^[^ ^41^ ^]^ As shown in Figure 7c, an optical 300‐fs laser pulse can switch the material from a neutral to an ionic state on a 500‐ps time scale. The self‐organized long‐range structural order is generated by strongly coupled electronic and structural changes, and the structure refinement before and after laser irradiation indicates a macroscopic ferroelectric reorganization. In the ferroelectric semiconductor Sn_2_P_2_S_6_, a phase transition initiated by laser radiation has been investigated via OPOP spectroscopy that takes place on the order of 10 ps, and the dynamics of electronic and soft phonon modes excitations can be used to interpret the structural phase transitions.^[^ ^97^, ^98^ ^]^ In the non‐equilibrium route, new phase states of matter are created. As shown in Figure 7d, a supercrystal phase induced by optical are observed from X‐ray data, which is converted from a two phase mixture of ferroelectric‐ferroelastic *a*_1_–*a*_2_ domains and polarization vortices in the atomic‐scale PbTiO_3_ (PTO)/STO superlattices.^[^ ^96^ ^]^ The microscopic mechanism of photo‐induced charge‐stabilized supercrystal consists of ultrafast carriers and lattice dynamics that has not been created via equilibrium routes and can be erased by heating. In another cases of STO samples, the host photoinduced metastable collective states, or “hidden phases” are observed via OPSP and TPSP spectroscopy.^[^ ^40^, ^94^ ^]^ These metastable polar phase and “hidden phase” are typically not accessible in equilibrium state, and may persist long after the removal of external stimuli. As the SHG process only exits in materials that lack inverse symmetry, the time delay‐independent total SHG intensity shown in Figure 7e presents a reliable reporter of a photoinduced phase with broken inversion symmetry in STO.^[^ ^40^ ^]^ The ultrafast resonant excitation of crystal lattice vibrations (phonons) plays key roles in reaching new phase, and the soft phonon mode serves as a collective reaction coordinate that the ions move from their initial positions toward their positions in the new phase.

### 4.2 Acoustic Phonon Excitations and Strain Propagation

**Figure 8:**
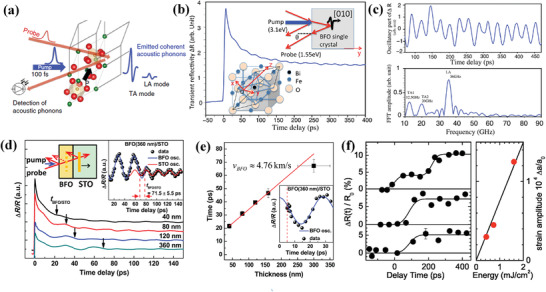
a) Principle of the ultrafast optical pump‐probe experiments. Reproduced with permission.^[^ ^71^ ^]^ Copyright 2014, Nature Publishing Group. b) Time resolved optical reflectivity of a [010] BFO single crystal. c) Brillouins oscillations detected in [109]‐pseudocubic direction of BFO single crystal, and corresponding fast Fourier transform revealing the three acoustic modes. b,c) Reproduced with permission.^[^ ^100^ ^]^ Copyright 2012, American Institute of Physics. d) The ∆*R*/*R* on (110) BFO thin films with various thicknesses. e) The thickness‐dependent strain pulse propagating time through the interface between BFO and STO. d,e) Reproduced with permission.^[^ ^53^ ^]^ Copyright 2012, American Institute of Physics. f) Left panel: Measured angle‐integrated diffraction intensity of the (004) reflection of STO for different pump fluences of (top) 1.6 mJ cm^−2^, (middle) 0.74 mJ cm^−2^ and (bottom) 0.43 mJ cm^−2^. Right panel: Measured strain amplitude (circles) versus excitation fluence together with linear fit. Reproduced with permission.^[^ ^102^ ^]^ Copyright 2006, American Physical Society.

The ultrafast acoustic phonon excitations in ferroelectrics are also efficiently photogenerated and photodetected by time‐resolved pump‐probe spectroscopy,^[^ ^99^ ^]^ since they play an important role in ferroelectric‐based electroacoustic and acoustooptic devices. The schematic diagram of as‐generated coherent gigahertz (GHz) acoustic phonons of longitudinal acoustic (LA) as well as transverse acoustic (TA) modes in BFO samples is shown in **Figure** **8**a.^[^ ^71^ ^]^ The femtosecond pump light can create electron‐hole pairs in the narrow band gap of BFO, which initiates the screening of internal polarization and leads to the emission of coherent LA and TA acoustic phonons. As shown in Figure 8b, the propagation of photogenerated acoustic phonons is contained by transient reflectivity signals ∆*R*/*R* via OPOP spectroscopy with clearly long‐living oscillations.^[^ ^100^ ^]^ By subtracting baseline of transient ∆*R* reflectivity signals and appropriating fast Fourier transformation, Figure 8c presents three acoustic modes at TA1, TA2, and LA corresponding to 12.5, 20, and 36 GHz. The sound velocity of these three acoustic phonons are calculated to be 1720–1920, 2750–3070, and 4965–5538 m s^−1^, respectively in BFO single crystal. In polycrystalline BFO ceramics, extremely efficient opto‐acoustic coherent shear phonons are obtained, which can be tailored by crystallographic orientations.^[^ ^71^ ^]^ Such a giant amplitude of shear mode results in a largest intensity ratio GHz transverse versus the LA waves. In the birefringent ferroelectrics BFO and LNO, all‐optical ultrafast mechanism mediated by acousto–optic interaction are revealed, which show that the GHz coherent acoustic phonons can induce a conversion of light polarization.^[^ ^101^ ^]^ As a direct consequence, an all‐optical device for ultrafast manipulation of the light information can be developed by using short laser pulse for launching in ferroelectric media coherent GHz acoustic phonons capable to modify light polarization.

As the generated acoustic phonons would create a strain field that propagates and modulates the refractive index of a medium, the ultrafast strain propagation can be studied via OPOP spectroscopy. In BFO thin films, the oscillated feature of ∆*R*/*R* curve associated with strain pulse is measured in Figure 8d, which can be phenomenologically described as:^[^ ^53^ ^]^

(3) $$\begin{array}{ccc} \frac{\Delta R}{R} & = & A_{1}\exp\left(-\frac{t}{\tau_{1}}\right)+A_{2}\exp\left(-\frac{t}{\tau_{2}}\right)+A_{3}\\ & & +\;A_{0}\exp\left(-\frac{t}{\tau_{0}}\right)\cos\left(\frac{2\pi t}{T}+\phi \right) \end{array}$$

The 1st term on the right‐hand represents the decay of excited electrons. The 2nd term represents the *d*‐*d* transitions of Fe^3+^ ions, which are relaxed through the spin‐orbit coupling and octahedral distortion. The 3rd term describes the energy loss during transition from the hot spot to ambient environment within microseconds. The last term denotes the oscillation component associated with strain pulse propagation, and *τ*_0_, *T*, and *ϕ* refer to damping time, period, and initial phase of the oscillation, respectively. Based on the Equation (3), the fitted period is ≈29.3 ps, and the strain pulse velocity in [110] direction of BFO film is found to be 4.88 km s^−1^. As shown in Figure 8e, the calculated strain pulse velocity is close to velocity (4.76 km s^−1^) that is obtained from thickness‐dependent *t*_BFO_/_STO_ measurements. The ultrafast anisotropic photostriction is found to be mainly derived from the optical rectification effect, which opens pathways to design ultrafast device with multifunctionality.

The dynamics of strain propagation in ferroelectric are also investigated using OPXP spectroscopy, which can provide direct insights into the generation and propagation of strain by monitoring atomic motions via the changes of Bragg diffraction pattern. As the strain propagates at the speed of 8 km s^−1^, the nanostructured perovskite with a thickness of *d*≤100 nm possesses the relevant time scale of strain transients in the order of tens of picoseconds.^[^ ^102^ ^]^ In the case of PZT/SrRuO_3_ (SRO) perovskite heterostructure deposited on STO substrates,^[^ ^102^ ^]^ the strain propagation is presented by a two‐step time evolution, which originates from the interference of X‐rays diffracted from the strained and unstrained parts of sample. As shown in Figure 8f, the time‐dependent evolution of the intensity of (004) Bragg reflection from STO substrate strongly depends on the optical pump fluences. The signals clearly grow in two steps with an increase in reflectivity at a higher excitation (bottom panel of Figure 8f), whereas only one step is found for weaker excitation. The significant changes in time evolution with respect to pump fluences indicate that the femtosecond displacive phonon excitation can launch acoustic strain waves propagating into the STO substrate. Analysis by dynamical XRD theory, the ultrafast transient strain is determined as small as ∆*a*/*a*_0_ = 2 × 10^−5^. In another case of PZT thin film on SRO metal and STO substrate with mosaic structure, the imperfect mosaic structures can lead to a coupling of excited out‐of‐plane expansion that expands in‐plane lattice dynamics (atoms to start moving) on a picosecond time scale.^[^ ^103^ ^]^ For the BFO thin films, the giant optical enhancement in strain gradient 10^5^–10^6^ m^−1^ can last up a few nanosecond, which is dependence on film thickness and attributed to a piezoelectric effect driven by a transient screening field mediated by excitons.^[^ ^104^ ^]^

## 5 Spin Dynamics of Multiferroics

**Figure 9:**
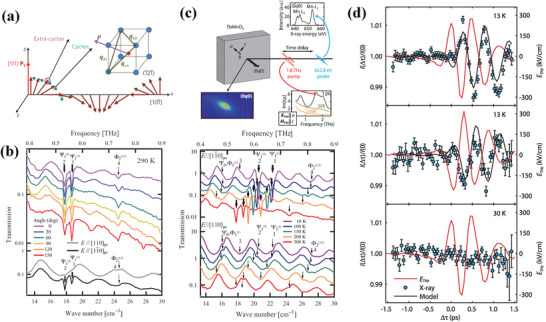
a) Spin cycloid ground state along with its low‐energy excitations when the ferroelectric polarization *P*_1_ points along the [110] direction in BFO. Reproduced with permission.^[^ ^106^ ^]^ Copyright 2010, Nature Publishing Group. b) Left panel: Polarization dependent THz transmission spectra of (111)_pc_‐oriented (upper) and (001)_pc_‐oriented (lower) BFO crystals at room temperature. Right panel: Temperature dependent THz transmission spectra of (001)_pc_‐oriented BFO for THz polarizations along the $\left[1\bar{1}0\right]$_pc_ (upper) and the [109]_pc_ (lower) axes. Reproduced with permission.^[^ ^108^ ^]^ Copyright 2016, American Physical Society. c) Schematic of a THz pulse resonant with the strongest electromagnon excites spin motion in the TbMnO_3_ sample. d) Time‐dependent behavior. The (0*q*0) peak of TbMnO_3_ (blue symbols, left axis) compared with the electric field *E*_THz_ (red solid line, right axis) as a function of the time delay. (Top and Middle panels). Bottom panel: The response in non‐multiferroic phase (*T* = 30 K). c,d) Reproduced with permission.^[^ ^39^ ^]^ Copyright 2014, American Association for the Advancement of Science.

Thanks to the coexistence of ferroelectric and ferromagnetic order, multiferroics provide potential applications through electric field controlled magnetic order or magnetic field controlled ferroelectric order.^[^ ^25^, ^83^ ^]^ The magnetoelectric coupling between ferroelectric and magnetic results in a mixed excitations of phonons and magnons, which gives magnon‐likes modes excited by electric field or phonon‐like modes excited by magnetic fields.^[^ ^105^ ^]^ As direct excitation of spin always occurs in the THz frequency range, the magnons and electric‐dipole active spin excitations (called electromagnons) can be easily tuned to resonance by an intense few‐cycle THz light pulse with derived spin dynamics.^[^ ^39^ ^]^ In the case of room‐temperature multiferroic BFO, the cycloidal spin planes are determined by ferroelectric polarization *P* and antiferromagnetic cycloid wave vectors *q* in **Figure** **9**a.^[^ ^106^ ^]^ The normal magnon modes of BFO can be separated into two modes of in‐plane modes (*Φ_±n_*) and out‐of‐plane modes (*Ψ_±n_*), which is below 70 cm^−1^ of the frequency of the lowest phonon mode.^[^ ^81^ ^]^ The frequency and dispersion of magnon resonance are determined by the strength of antiferromagnetic exchange and magnetic anisotropy,^[^ ^107^ ^]^ where the magnetic dipole selection results in the assignment of most prominent resonances during THz polarization. Based on this, the temperature dependence or polarization dependence of THz transmission spectra are observed in BFO samples via TPTP spectroscopy.^[^ ^81^, ^107^, ^108^ ^]^ In a (111)*_pc_*‐oriented BFO single crystal, two major magnon absorption signals of Ψ_1_^(2)^ and Ψ_1_^(1)^ are observed, which do not exhibit any dependence on THz polarization (left panel in Figure 9b).^[^ ^108^ ^]^ This results indicate that the magnetic domains are not aligned in one of the three equivalent allowed antiferromagnetic cycloid directions. In a (001)*_pc_*‐oriented BFO single crystal, the absorption of Ψ_1_^(2)^ and Ψ_1_^(1)^ only appears at the THz polarization along the$\left[1\bar{1}0\right]$_pc_ axis, and the polarization dependence does not change even after annealing the sample at a temperature above the Neel point (right panel in Figure 9b). This selection rules are similar to the *E*‐mode phonons of BFO, which illustrate that the magnon‐phonon coupling (a coupling between magnetic and lattice excitations) is strong enough to govern the polarization dependence of magnon absorptions. Though the magnetoelectric coupling would shift their excitation frequencies with respects to the bare magnon or phonon frequencies, the frequencies of magnetic resonances and electromagnons are coincident within experimental resolution in BFO samples for their small energy shifts. According to the measured several absorption resonances at various temperatures, the strongest absorption lines between 18 and 30 cm^−1^ are ascribed to a combined magnon and electromagnon response of Ψ_3_, Φ_3_, and Φ_4_ modes in BFO crystal.^[^ ^107^ ^]^ The main difference between magnon and electromagnon modes is that the electromagnons would contribute to the dielectric susceptibility like phonon modes, but the magnons would not. Based on the coupling weight of electromagnons on dielectric constant, the colossal electromagnon are observed at polar‐antipolar and antiferromagnetic‐ferromagnetic phase boundaries in the Nd dope BFO nanoparticles.^[^ ^83^ ^]^

In another representative signal‐phase multiferroics TbMnO_3_, as schematically illustrated in Figure 9c, the spin rotations driven by THz pulse are observed by TPXP spectroscopy.^[^ ^39^ ^]^ The spin dynamics are extracted from the intensity of (0*q*0) diffraction peak as a function of pump‐probe delay time at different temperatures in Figure 9d. When the TbMnO_3_ is deep in multiferroic phase at 13 K, the larger modulation is observed. The first maximum delay between pump and X‐ray traces is ≈250 fs, which corresponds to about half of a single oscillation cycle. When the TbMnO_3_ is in the non‐multiferroic phase at 30 K, the oscillation in the peak intensity after the pump is strongly suppressed. The temperature dependence results provide strong evidence that the THz induced spin motion is correlated with the presence of multiferroicity. The observed maximum change of peak intensity appears ≈1.35% ± 0.12% corresponding to an amplitude of spin cycloid plane rotation that equal to 4.2° ±0.4°. It suggests that a THz pulse with amplitude of 1 to 2 MV cm^−1^ could lead to spin‐cycloid rotations in the order of 90° by simple linear extrapolation. Another case is in the multiferroic heterostructure of Ba_0.1_Sr_0.9_TiO_3_/La_0.7_Ca_0.3_MnO_3_ (BSTO/LCMO),^[^ ^70^ ^]^ the coupling between electric and magnetic order is controlled on ultrafast timescales using OPSP spectroscopy with the advantage of phase‐sensitive in SHG. The coupling between electric and magnetic order is induced within tens of picoseconds, which can be mediated through elastic coupling between BSTO and LCMO layers.

## 6 Photo‐Induced Electronic Excitation Dynamics of Ferroelectrics

**Figure 10:**
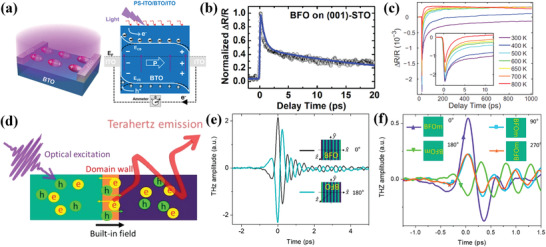
a) Schematic illustration of the ferroelectric based optoelectronic device architectures. Reproduced with permission.^[^ ^109^ ^]^ Copyright 2018, Elsevier BV. b) Normalized temporal evolution of differential reflectivity of tetragonal BFO films on (001)‐STO. Reproduced with permission.^[^ ^54^ ^]^ Copyright 2012, American Institute of Physics. c) Photoinduced transient reflectivity change of BFO signal crystal at various temperatures measured at 3.18 eV. Reproduced with permission.^[^ ^57^ ^]^ Copyright 2012, American Institute of Physics. d) THz emission mechanism in the periodic stripe domain sample. e) Time‐domain THz transients for the sample in 0° and 180° orientations, where the domain walls lie along +*ŷ*, perpendicular to the *xz* plane with the net in‐plane polarization along ‐*x̂* in 0° orientation. f) Radiated THz transients for different azimuthal orientations of the monodomain (110) BFO sample. d‐f) Reproduced with permission.^[^ ^110^ ^]^ Copyright 2020, American Chemical Society.

Photovoltaic effect in ferroelectrics is another fascinating topic, which has received considerable attention due to its special features of polarization‐dependent photocurrent and above band‐gap photovoltage.^[^ ^109^ ^]^ Upon the appropriate wavelengths of light, the photo‐induced electron‐hole pairs can be excited (left panel in **Figure** **10**a), which can be separated by the internal electric field in poled ferroelectrics due to the photovoltaic effect (right panel in Figure 10a). These separated photogenerated carries can lead to the generation of current/voltage signals in the external circuit, which provides a wonderful wide range of ferroelectric based optoelectronic applications (i.e., energy conversion and information storage). Recently, a simple OPOP spectroscopy is being widely used to study the dynamics of photogenerated carriers. As the photogenerated carriers are excited at the photon energy larger than the bandgap of a material, the wavelength of pump laser is important. In the case of BFO films (*E*_g_ ≈ 2.6–2.8 eV),^[^ ^54^ ^]^ the ultrafast carrier dynamics and radiative recombination are observed by measuring the typical ∆*R*/*R*, under the illumination of 400 nm femtosecond laser pulses (photon energy 3.1 eV). From the ∆*R*/*R* curves in Figure 10b, the swift rise at *t* = 0 is originated from electronic excitations. The fast process (≈1 ps) corresponds to the scattering of electrons with lattice‐vibration modes (electro‐phonon interaction), while the subsequent slow process (≈tens of ps) is due to the spin‐lattice thermalization. Both fast and slow processes are consistent with the physical process shown in Equation (3). As the obtained strength of electro‐phonon interaction exhibits structural dependent, the relaxation time constants *τ*_*e* − *ph*_ in tetragonal BFO films is found to be larger than the rhombohedral counterparts, for the enhanced structural strain and symmetry breaking in tetragonal BFO. In the BFO single crystal, three decay time constants, including fast time constant (1 ps), intermediate time constant (10–50 ps) and slow time constant (1–3 ns), are observed and fitted in Figure 10c. With increasing measurement temperature up to 800 K (Neel temperature is 640 K in BFO),^[^ ^57^ ^]^ the first fast time constant is found nearly constant, which indicates that the electron‐phonon coupling does not depend on temperature. Though the intermediate time constant becomes faster at higher temperatures, no abrupt change is observed in the measured dynamics across *T*_N_. It means that the second slow process of electron‐phonon coupling does not dramatically change with temperature, and the spin‐lattice relaxation are verified not playing a significant role in the observed dynamics. The temperature‐dependent second relaxation process are attributed to the photoexcited electrons leaving the conduction band, either through direct radiative recombination or through radiative recombination from other lower lying electronic states. The presented longer slow time delays (1–3 ns) are attributed to the combination of heat diffusion (positive component of the signal) and recombination of carriers (negative component of the signal).

The OPTP spectroscopy are also employed to study the ultrafast charge separation or strong electron‐phonon coupling of ferroelectrics.^[^ ^79^, ^111^ ^]^ In hybrid organic‐inorganic perovskite methylammonium lead iodide (CH_3_NH_3_PbI_3_, MAPI),^[^ ^79^ ^]^ the initial charge separation is demonstrated to be driven by diffusion instead of surface fields or intrinsic ferroelectricity based on the emitted THz pulses. The rapid initial cooling of hot carriers in MAPI is observed at 250 fs, and the excitation of the coherent lattice modes take place after this process persist for 3–4 ps. In the case of BFO films with spontaneously formed periodic stripe domains, the dominant photovoltaic mechanism of periodic stripe domain and domain wall mediated charge separation is disentangled and quantified. As shown Figure 10d, the above‐band gap 400 nm femtosecond pulses are used in BFO film for excitation with the emitted THz field.^[^ ^110^ ^]^ In Figure 10e,f, the emitted THz transients from the stripe domain and monodomain BFO films are measured at different azimuthal orientations. The completely flipped polarity of the THz field at two azimuthal angles of 0° and 180° in Figure 10e indicates that the transient current giving rise to the emitted THz fields must be an in‐plane current in the stripe domain BFO films. A clearly larger absolute amplitude is exhibited in Figure 10f in the orientations of 0° comparing 180°. It suggests that the THz emission stems in the monodomain BFO sample are from the in‐plane and out‐of‐plane currents in the 0° and 180° orientations. By comparing the experimental amplitudes of transient currents obtained from the radiated THz signals and theoretical calculated shift currents, it shows strong evidence that the current density in stripe domain BFO is dominated by the charge separation across the domain walls that associated with the density of photogenerated carriers. The photovoltaic effect in monodomain BFO sample is governed by the response of shift current, which is associated with non‐centrosymmetry of the crystal. As the peak current amplitude driven by the charge separation at the domain walls is found to be two orders of magnitude higher than the bulk shift current response, the domain walls render a prominent role as nanoscale junctions to efficiently separate photogenerated charges in the stripe domain BFO films.

## 7 Conclusions

In summary, the major advances in the utilization of ultrafast time‐resolved pump‐probe spectroscopy to reveal dynamics of ferroelectrics, including polarization, lattice, spin, photo‐induced electronic excitation in the time range of femtosecond to nanosecond are reviewed. In ferroelectrics, the ultrafast polarization reversal originates from intrinsic atomic response, typically occurring in sub‐picosecond and picosecond time scale, while domain dynamics occurs in sub‐nanosecond to nanosecond time scale. The ultrafast spin excitation, and coupled magnetic as well as ferroelectric order also occur in the sub‐picosecond to picosecond range. The lattice dynamics, originating from phonon‐phonon interactions, occur in the range of a few picoseconds to hundreds of picoseconds. The sound velocity of acoustic phonons or speed of strain propagation occur in the range of several kms^−1^. The electron‐electron interactions occur in a femtosecond, and the electron‐phonon and spin‐lattice coupling occur in a few picoseconds to tens of picoseconds, whereas the recombination of carriers occur in several nanoseconds. The time‐resolved pump‐probe spectroscopy undoubtedly have their own uniqueness and application focus in ferroelectrics, which are summarized in **Table** **1**. The THz spectroscopy is a powerful and desirable tool to investigate polarization dynamics in ferroelectric and spin dynamics in multiferroics. The X‐ray probe spectroscopy provides useful insights into the dynamics of ferroelectric related to lattices or crystal changes, while the optical pump spectroscopy focuses on the electronic excitations dynamics. It is worth emphasizing that the fundamental understanding of the ultrafast dynamics of ferroelectric is important for the rapid development of high‐speed and small‐size ferroelectric based devices.

**Table 1:** The summary of literature survey on the investigation of ultrafast dynamics phenomenon of in ferroelectrics by pump‐probe technology

Physical effects/Excitation		Technique	Material
Polarization dynamics	polarization dynamics, polarization reversal dynamics, polarization modulation, domain/domain wall dynamics, photoassisted polarization switching	OPOP	BFO,^[^ ^42^, ^52^, ^72^, ^86^, ^88^ ^]^ BSTO,^[^ ^89^, ^90^ ^]^ BTO,^[^ ^38^ ^]^
		THz emission spectroscopy	*α*‐(ET)_2_I_3_,^[^ ^73^ ^]^
		TPOP	TTF‐CA,^[^ ^51^, ^92^ ^]^ LNO,^[^ ^37^ ^]^
		TPXP	croconic acid,^[^ ^93^ ^]^
		OPSP	
		THz pump SHG probe	
		OPXP	
Lattice dynamics	lattice vibration/ optical phonon excitation, acoustic phonon, acousto‐optic conversion, ultrafast photoinduced strain, phase transition, photovoltaic response, electron‐lattice coupling, electron‐phonon coupling, phonon‐polariton coupling, lattice‐polarization coupling	THz time domain spectroscopy	BTO,^[^ ^95^, ^112^ ^]^ PZT,^[^ ^102^, ^103^ ^]^ STO,^[^ ^40^, ^43^, ^68^, ^113^, ^114^ ^]^ BFO^[^ ^53^, ^71^, ^82^, ^100^, ^101^, ^104^ ^]^
		OPOP	LNO,^[^ ^55^, ^101^ ^]^ PTO,^[^ ^94^, ^96^, ^115^ ^]^ TTF‐CA,^[^ ^41^ ^]^ Sn_2_P_2_S_6_^[^ ^97^ ^]^
		OPSP	
		THz pump SHG probe	
		OPXP	
		TPOP	
		THz emission spectroscopy	
Spin dynamics	magnetic/spin excitation, electric‐magnetic coupling, magnon‐phonon coupling, electromagnon	OPOP	BFO,^[^ ^81^, ^83^, ^105^, ^107^, ^108^ ^]^ BSTO/LCMO,^[^ ^70^ ^]^
		THz time domain spectroscopy	TbMnO_3_^[^ ^39^ ^]^
		OPSP	
		TPXP	
Photogenerated carrier dynamics	electronic excitation, carrier dynamics/relaxation, radiative recombination, charge separation	OPOP	BFO,^[^ ^54^, ^57^, ^110^ ^]^ CH_3_NH_3_PbI_3_^[^ ^79^ ^]^
		THz emission spectroscopy	

## 8 Outlook

For ferroelectrics, the most striking feature of time‐resolved pump‐probe spectroscopy is the rapidly collective coherent control over material structure. The phase transitions from paraelectric to ferroelectricity, or “hidden phases” that are typically not accessible on equilibrium phase diagrams, can be effectively achieved by pump‐probe techniques. The pump‐probe techniques even give rise to an additional degree of freedom, and in turn provide potential approaches to nondestructive readout in nonvolatile ferroelectric memory. The pump‐probe techniques are being developed for higher temporal resolution, higher spatial resolution and diverse functions. Integrating multi‐field external stimuli of strain, electric, magnetic, and thermal fields in the time‐resolved pump‐probe spectroscopy can effectively expand their application prospects and scope. It is expected to reveal the microscopic mechanism of multi‐field coupling in ferroelectrics and other functional materials, and realize ultrafast multi‐function control in the further devices. For example, ultrafast time‐resolved pump‐probe force microscopy with nanoscale resolution^[^ ^116^ ^]^ and optical pump‐probe scanning tunneling microscopy^[^ ^117^, ^118^, ^119^ ^]^ with enabled visualization of ultrafast carrier dynamics on single‐atomic level have been developed. The pump‐probe techniques may also offer an additional nondestructive functionality in ultrafast device design. Ultrafast data reading, enhanced storage capacity, and laser‐assisted electronic data recording in ferroelectric memories may be materialized in further.

## Conflict of Interest

The authors declare no conflict of interest.

## References

[1] P. Zhang , F. Wang , M. Yu , X. Zhuang , X. Feng , Chem. Soc. Rev. 2018, 47, 7426. 30206606 10.1039/c8cs00561c

[2] F. An , K. Qu , G. Zhong , Y. Dong , W. Ming , M. Zi , Z. Liu , Y. Wang , B. Qi , Z. Ding , J. Xu , Z. Luo , X. Gao , S. Xie , P. Gao , J. Li , Adv. Funct. Mater. 2020, 30, 2003495.

[3] C. Ren , G. Zhong , Q. Xiao , C. Tan , M. Feng , X. Zhong , F. An , J. Wang , M. Zi , M. Tang , Y. Tang , T. Jia , J. Li , Adv. Funct. Mater. 2019, 30, 1906131.

[4] G. Zhong , M. Zi , C. Ren , Q. Xiao , M. Tang , L. Wei , F. An , S. Xie , J. Wang , X. Zhong , M. Huang , J. Li , Appl. Phys. Lett. 2020, 117, 092903.

[5] P. S. Peercy , Nature 2000, 406, 1023. 10984060 10.1038/35023223

[6] M. J. Madou , Fundamentals of Microfabrication: The Science of Miniaturization, CRC Press, Boca Raton, FL 2018.

[7] J. F. Scott , Science 2007, 315, 954. 17303745 10.1126/science.1129564

[8] G. Dong , S. Li , M. Yao , Z. Zhou , Y. Zhang , X. Han , Z. Luo , J. Yao , B. Peng , Z. Hu , H. Huang , T. Jia , J. Li , W. Ren , Z. Ye , X. Ding , J. Sun , C. Nan , L. Chen , J. Li , M. Liu , Science 2019, 366, 475. 31649196 10.1126/science.aay7221

[9] Y. You , W. Liao , D. Zhao , H. Ye , Y. Zhang , Q. Zhou , X. Niu , J. Wang , P. Li , D. Fu , Z. Wang , S. Gao , K. Yang , J. Liu , J. Li , Y. Yan , R. Xiong , Science 2017, 357, 306. 28729511 10.1126/science.aai8535

[10] P. Sharma , Q. Zhang , D. Sando , C. Lei , Y. Liu , J. Li , V. Nagarajan , J. Seidel , Sci. Adv. 2017, 3, e1700512. 28691100 10.1126/sciadv.1700512 PMC5482552

[11] Z. Li , Y. Wang , G. Tian , P. Li , L. Zhao , F. Zhang , J. Yao , H. Fan , X. Song , D. Chen , Z. Fan , M. Qin , M. Zeng , Z. Zhang , X. Lu , S. Hu , C. Lei , Q. Zhu , J. Li , X. Gao , J. Liu , Sci. Adv. 2017, 3, e1700919. 28835925 10.1126/sciadv.1700919 PMC5562417

[12] M. Ye , S. Hu , Y. Zhu , Y. Zhang , S. Ke , L. Xie , Y. Zhang , S. Hu , D. Zhang , Z. Luo , M. Gu , J. He , P. Zhang , W. Zhang , L. Chen , Nano Lett. 2021, 21, 144. 33306405 10.1021/acs.nanolett.0c03417

[13] J. Wang , B. Wang , L. Chen , Annu. Rev. Mater. Res. 2019, 49, 127.

[14] P. Chen , X. Zhong , J. A. Zorn , M. Li , Y. Sun , A. Y. Abid , C. Ren , Y. Li , X. Li , X. Ma , J. Wang , K. Liu , Z. Xu , C. Tan , L. Chen , P. Gao , X. Bai , Nat. Commun. 2020, 11, 1840. 32296053 10.1038/s41467-020-15616-y PMC7160157

[15] X. Li , C. Tan , C. Liu , P. Gao , Y. Sun , P. Chen , M. Li , L. Liao , R. Zhu , J. Wang , Y. Zhao , L. Wang , Z. Xu , K. Liu , X. Zhong , J. Wang , X. Bai , Proc. Natl. Acad. Sci. U. S. A. 2020, 117, 18954. 32709747 10.1073/pnas.2007248117 PMC7430988

[16] X. L. Zhong , J. B. Wang , M. Liao , G. J. Huang , S. H. Xie , Y. C. Zhou , Y. Qiao , J. P. He , Appl. Phys. Lett. 2007, 90, 152903.

[17] Y. Sun , A. Abid , C. Tan , C. Ren , M. Li , N. Li , P. Chen , Y. Li , J. Zhang , X. Zhong , J. Wang , M. Liao , K. Liu , X. Bai , Y. Zhou , D. Yu , P. Gao , Sci. Adv. 2019, 5, eaav4355. 31700996 10.1126/sciadv.aav4355 PMC6824850

[18] Z. M. Zhao , F. An , F. G. Tian , Y. Zhang , Q. F. Zhu , L. Chen , G. K. Zhong , S. H. Xie , J. Appl. Phys. 2020, 127, 134101.

[19] C. Liu , F. An , P. S. M. Gharavi , Q. Lu , J. Zha , C. Chen , L. Wang , X. Zhan , Z. Xu , Y. Zhang , K. Qu , J. Yao , Y. Ou , Z. Zhao , X. Zhong , D. Zhang , N. Valanoor , L. Chen , T. Zhu , D. Chen , X. Zhai , P. Gao , T. Jia , S. Xie , G. Zhong , Natl. Sci. Rev. 2020, 7, 84. 34692020 10.1093/nsr/nwz143 PMC8289034

[20] G. H. Haertling , J. Am. Ceram. Soc. 1999, 82, 797.

[21] Y. Zhang , Q. Guo , S. Zheng , X. Zhong , G. Zhong , D. Zhang , C. Ren , C. Tan , Z. Lu , Y. Zhang , Y. Tang , J. Wang , J. Yuan , J. Mater. Chem. C 2018, 6, 11679.

[22] L. Chen , Y. Zhang , Q. Guo , D. Zhang , X. Zhong , J. Yuan , Appl. Phys. Lett. 2014, 105, 112903.

[23] S. Li , X. L. Zhong , G. H. Cheng , X. Liu , Y. Zhang , J. B. Wang , H. J. Song , C. B. Tan , B. Li , Appl. Phys. Lett. 2015, 106, 142904.

[24] G. Zhong , F. An , Y. Bitla , J. Wang , X. Zhong , J. Yu , W. Gao , Y. Zhang , C. Tan , Y. Ou , J. Jiang , Y. H. Hsieh , X. Pan , S. Xie , Y. H. Chu , J. Li , ACS Nano 2018, 12, 9558. 30138564 10.1021/acsnano.8b05284

[25] G. Zhong , Y. Bitla , J. Wang , X. Zhong , F. An , Y.‐Y. Chin , Y. Zhang , W. Gao , Y. Zhang , A. Eshghinejad , E. N. Esfahani , Q. Zhu , C. Tan , X. Meng , H.‐J. Lin , X. Pan , S. Xie , Y.‐H. Chu , J. Li , Acta Mater. 2018, 145, 488.

[26] C.‐W. Nan , Phys. Rev. B 1994, 50, 6082. 10.1103/physrevb.50.6082 9976980

[27] T. Haider , Int. J. Electromagn. Appl. 2017, 7, 17.

[28] K. Uchino , Ferroelectric Devices, CRC Press, Boca Raton, FL 2010.

[29] H. Huang , J. F. Scott , Ferroelectric Materials for Energy Applications, Wiley‐VCH, Weinheim 2018.

[30] K. Wang , J. Ouyang , M. Wuttig , Y. Zhao , H. Cheng , Y. Zhang , R. Su , J. Yan , X. Zhong , F. Zeng , Adv. Energy Mater. 2020, 10, 2001778.

[31] M. Pashkevich , Ultrafast Light‐Induced Magnetization Dynamics in Co/Garnet Heterostructures, University of Bialystok, XXXX 2015.

[32] S. O. Sayedaghaee , C. Paillard , S. Prosandeev , B. Xu , L. Bellaiche , npj Comput. Mater. 2020, 6, 60.

[33] C. Jia , S. Onoda , N. Nagaosa , J. H. Han , Phys. Rev. B 2007, 76, 144424.

[34] H. Palneedi , V. Annapureddy , S. Priya , J. Ryu , Actuators 2016, 5, 9.

[35] M. M. Vopson , J. Phys. D: Appl. Phys. 2013, 46, 345304.

[36] C. Schmising , M. Bargheer , M. Kiel , N. Zhavoronkov , M. Woerner , T. Elsaesser , I. Vrejoiu , D. Hesse , M. Alexe , Phys. Rev. Lett. 2007, 98, 257601. 17678054 10.1103/PhysRevLett.98.257601

[37] R. Mankowsky , A. Hoegen , M. Forst , A. Cavalleri , Phys. Rev. Lett. 2017, 118, 197601. 28548509 10.1103/PhysRevLett.118.197601

[38] F. Chen , Y. Zhu , S. Liu , Y. Qi , H. Y. Hwang , N. C. Brandt , J. Lu , F. Quirin , H. Enquist , P. Zalden , T. Hu , J. Goodfellow , M. J. Sher , M. C. Hoffmann , D. Zhu , H. Lemke , J. Glownia , M. Chollet , A. R. Damodaran , J. Park , Z. Cai , I. W. Jung , M. J. Highland , D. A. Walko , J. W. Freeland , P. G. Evans , A. Vailionis , J. Larsson , K. A. Nelson , A. M. Rappe , K. Sokolowski‐Tinten , L. W. Martin , H. Wen , A. M. Lindenberg , Phys. Rev. B 2016, 94, 180104.

[39] T. Kubacka , J. A. Johnson , M. C. Hoffmann , C. Vicario , S. de Jong , P. Beaud , S. Grübel , S.‐W. Huang , L. Huber , L. Patthey , Y.‐D. Chuang , J. J. Turner , G. L. Dakovski , W.‐S. Lee , M. P. Minitti , W. Schlotter , R. G. Moore , C. P. Hauri , S. M. Koohpayeh , V. Scagnoli , G. Ingold , S. L. Johnson , U. Staub , Science 2014, 343, 1333. 24603154 10.1126/science.1242862

[40] T. F. Nova , A. S. Disa , M. Fechner1 , A. Cavalleri , Science 2019, 364, 1075. 31197010 10.1126/science.aaw4911

[41] E. Collet , M.‐H. L. Cailleau , M. B.‐L. Cointe , H. Cailleau , M. Wulff , T. Luty , S. Koshihara , M. Meyer , L. Toupet , P. Rabiller , S. Techert , Science 2003, 300, 612. 12714737 10.1126/science.1082001

[42] D. Rana , I. Kawayama , K. Mavani , K. Takahashi , H. Murakami , M. Tonouchi , Adv. Mater. 2009, 21, 2881.

[43] I. Katayama , H. Aoki , J. Takeda , H. Shimosato , M. Ashida , R. Kinjo , I. Kawayama , M. Tonouchi , M. Nagai , K. Tanaka , Phys. Rev. Lett. 2012, 108, 097401. 22463665 10.1103/PhysRevLett.108.097401

[44] J. Cabanillas‐Gonzalez , G. Grancini , G. Lanzani , Adv. Mater. 2011, 23, 5468. 22020959 10.1002/adma.201102015

[45] O. Synnergren , Time‐Resolved X‐Ray Diffraction Studies of Phonons and Phase Transitions, Lund University, Department of Physics, XXXX 2005.

[46] J. Zhu , X. Wu , D. M. Lattery , W. Zheng , X. Wang , Nanoscale Microscale Thermophys. Eng. 2017, 21, 177.

[47] R. D. Averitt , A. J. Taylor , J. Phys.: Condens. Matter 2002, 14, R1357.

[48] J. Shah , Ultrafast Spectroscopy of Semiconductors and Semiconductor Nanostructures, Springer Science & Business Media, New York 2013.

[49] C. L. Yang , J. Dai , W. K. Ge , X. Cui , Appl. Phys. Lett. 2010, 96, 152109.

[50] T. Kampfrath , K. Tanaka , K. A. Nelson , Nat. Photonics 2013, 7, 680.

[51] T. Miyamoto , H. Yada , H. Yamakawa , H. Okamoto , Nat. Commun. 2013, 4, 2586. 24131938 10.1038/ncomms3586 PMC3826650

[52] K. Takahashi , M. Tonouchi , Appl. Phys. Lett. 2007, 90, 052908.

[53] L. Y. Chen , J. C. Yang , C. W. Luo , C. W. Laing , K. H. Wu , J. Y. Lin , T. M. Uen , J. Y. Juang , Y. H. Chu , T. Kobayashi , Appl. Phys. Lett. 2012, 101, 041902.

[54] Z. Jin , Y. Xu , Z. Zhang , G. Li , X. Lin , G. Ma , Z. Cheng , X. Wang , Appl. Phys. Lett. 2012, 100, 071105.

[55] T. Kuribayashi , T. Motoyama , Y. Arashida , I. Katayama , J. Takeda , J. Appl. Phys. 2018, 123, 174103.

[56] S.‐T. Lou , F. M. Zimmermann , R. A. Bartynski , N. Hur , S.‐W. Cheong , Phys. Rev. B 2009, 79, 214301.

[57] Y. M. Sheu , S. A. Trugman , Y. S. Park , S. Lee , H. T. Yi , S. W. Cheong , Q. X. Jia , A. J. Taylor , R. P. Prasankumar , Appl. Phys. Lett. 2012, 100, 242904.

[58] M. Bass , Handbook of Optics: Optical Properties of Materials, Nonlinear Optics, Quantum Optics, McGraw Hill Professional, New York 2009.

[59] M. Vrakking , T. Schultz , Attosecond and XUV Physics: Ultrafast Dynamics and Spectroscopy, Wiley‐VCH, Weinheim 2014.

[60] J. Haddad , B. Bousquet , L. Canioni , P. Mounaix , TrAC, Trends Anal. Chem. 2013, 44, 98.

[61] P. Gilch , I. Hartl , Q. An , W. Zinth , J. Phys. Chem. A 2002, 106, 1647.

[62] A. Kirilyuk , A. V. Kimel , T. Rasing , Rev. Mod. Phys. 2010, 82, 2731.

[63] R. Gutzler , M. Garg , C. R. Ast , K. Kuhnke , K. Kern , Nat. Rev. Phys. 2021, 3, 441.

[64] D. Nicoletti , A. Cavalleri , Adv. Opt. Photonics 2016, 8, 401.

[65] J. Petzelt , S. Kamba , Ferroelectrics 2016, 503, 19.

[66] C. Kwamen , M. Rössle , W. Leitenberger , M. Alexe , M. Bargheer , Appl. Phys. Lett. 2019, 114, 162907.

[67] J. Hebling , K. Yeh , M. Hoffmann , K. Nelson , IEEE J. Sel. Top. Quantum Electron. 2008, 14, 345.

[68] M. Kozina , M. Fechner , P. Marsik , T. van Driel , J. M. Glownia , C. Bernhard , M. Radovic , D. Zhu , S. Bonetti , U. Staub , M. C. Hoffmann , Nat. Phys. 2019, 15, 387.

[69] Y. Zhu , F. Chen , J. Park , K. Sasikumar , B. Hu , A. R. Damodaran , I. W. Jung , M. J. Highland , Z. Cai , I. C. Tung , D. A. Walko , J. W. Freeland , L. W. Martin , S. K. R. S. Sankaranarayanan , P. G. Evans , A. M. Lindenberg , H. Wen , Phys. Rev. Mater. 2017, 1, 060601.

[70] Y. M. Sheu , S. A. Trugman , L. Yan , Q. X. Jia , A. J. Taylor , R. P. Prasankumar , Nat. Commun. 2014, 5, 5832. 25534775 10.1038/ncomms6832

[71] M. Lejman , G. Vaudel , I. C. Infante , P. Gemeiner , V. E. Gusev , B. Dkhil , P. Ruello , Nat. Commun. 2014, 5, 4301. 24980954 10.1038/ncomms5301

[72] K. Takahashi , N. Kida , M. Tonouchi , Phys. Rev. Lett. 2006, 96, 117402. 16605869 10.1103/PhysRevLett.96.117402

[73] H. Yamakawa , T. Miyamoto , T. Morimoto , H. Yada , Y. Kinoshita , M. Sotome , N. Kida , K. Yamamoto , K. Iwano , Y. Matsumoto , S. Watanabe , Y. Shimoi , M. Suda , H. M. Yamamoto , H. Mori , H. Okamoto , Sci. Rep. 2016, 6, 20571. 26864779 10.1038/srep20571 PMC4750076

[74] G. Luca , M. Rossell , J. Schaab , N. Viart , M. Fiebig , M. Trassin , Adv. Mater. 2017, 29, 1605145. 10.1002/adma.201605145 27936292

[75] J. Y. Chauleau , E. Haltz , C. Carretero , S. Fusil , M. Viret , Nat. Mater. 2017, 16, 803. 28481343 10.1038/nmat4899

[76] H. Yokota , J. Kaneshiro , Y. Uesu , Phys. Res. Int. 2012, 2012, 704634.

[77] J. Nordlander , N. Strkalj , M. Fiebig , M. Trassin , Appl. Sci. 2018, 8, 570.

[78] Y. Zhang , Y. Zhang , Q. Guo , X. Zhong , Y. Chu , H. Lu , G. Zhong , J. Jiang , C. Tan , M. Liao , Z. Lu , D. Zhang , J. Wang , J. Yuan , Y. Zhou , npj Comput. Mater. 2018, 4, 39.

[79] B. Guzelturk , R. A. Belisle , M. D. Smith , K. Bruening , R. Prasanna , Y. Yuan , V. Gopalan , C. J. Tassone , H. I. Karunadasa , M. D. McGehee , A. M. Lindenberg , Adv. Mater. 2018, 30, 1704737. 10.1002/adma.201704737 29359820

[80] M. Hangyo , M. Tani , T. Nagashima , Int. J. Infrared Millimeter Waves 2005, 26, 1661.

[81] U. Nagel , R. S. Fishman , T. Katuwal , H. Engelkamp , D. Talbayev , H. T. Yi , S. W. Cheong , T. Room , Phys. Rev. Lett. 2013, 110, 257201. 23829754 10.1103/PhysRevLett.110.257201

[82] S. Kamba , D. Nuzhnyy , M. Savinov , J. Šebek , J. Petzelt , J. Prokleška , R. Haumont , J. Kreisel , Phys. Rev. B 2007, 75, 024403.

[83] Y. Zhang , Y. Zhang , Q. Guo , D. Zhang , S. Zheng , M. Feng , X. Zhong , C. Tan , Z. Lu , J. Wang , P. Hou , Y. Zhou , J. Yuan , Phys. Chem. Chem. Phys. 2019, 21, 21381. 31531469 10.1039/c9cp04194j

[84] H. Akamatsu , Y. Yuan , V. A. Stoica , G. Stone , T. Yang , Z. Hong , S. Lei , Y. Zhu , R. C. Haislmaier , J. W. Freeland , L.‐Q. Chen , H. Wen , V. Gopalan , Phys. Rev. Lett. 2018, 120, 096101. 29547337 10.1103/PhysRevLett.120.096101

[85] H. Wen , M. J. Cherukara , M. V. Holt , Annu. Rev. Mater. Res. 2019, 49, 389.

[86] D. S. Rana , K. Takahashi , K. R. Mavani , I. Kawayama , H. Murakami , M. Tonouchi , Phys. Rev. B 2008, 77, 024105.

[87] D. S. Rana , K. Takahashi , K. R. Mavani , I. Kawayama , H. Murakami , M. Tonouchi , Appl. Phys. Lett. 2007, 91, 031909.

[88] F. Chen , J. Goodfellow , S. Liu , I. Grinberg , M. C. Hoffmann , A. R. Damodaran , Y. Zhu , P. Zalden , X. Zhang , I. Takeuchi , A. M. Rappe , L. W. Martin , H. Wen , A. M. Lindenberg , Adv. Mater. 2015, 27, 6371. 26389651 10.1002/adma.201502975

[89] K. A. Grishunin , N. A. Ilyin , N. E. Sherstyuk , E. D. Mishina , A. Kimel , V. M. Mukhortov , A. V. Ovchinnikov , O. V. Chefonov , M. B. Agranat , Sci. Rep. 2017, 7, 687. 28386081 10.1038/s41598-017-00704-9 PMC5429639

[90] E. Mishina , K. Grishunin , V. Bilyk , N. Sherstyuk , A. Sigov , V. Mukhortov , A. Ovchinnikov , A. Kimel , MRS Adv. 2018, 3, 1901.

[91] S. A. Denev , T. T. A. Lummen , E. Barnes , A. Kumar , V. Gopalan , D. J. Green , J. Am. Ceram. Soc. 2011, 94, 2699.

[92] T. Morimoto , T. Miyamoto , H. Yamakawa , T. Terashige , T. Ono , N. Kida , H. Okamoto , Phys. Rev. Lett. 2017, 118, 107602. 28339244 10.1103/PhysRevLett.118.107602

[93] M. Sotome , N. Kida , S. Horiuchi , H. Okamoto , Appl. Phys. Lett. 2014, 105, 041101.

[94] X. Li , T. Qiu , J. Zhang , E. Baldini , J. Lu , A. M. Rappe , K. A. Nelson , Science 2019, 364, 1079. 31197011 10.1126/science.aaw4913

[95] F. Wan , J. Han , Z. Zhu , Phys. Lett. A 2008, 372, 2137.

[96] V. A. Stoica , N. Laanait , C. Dai , Z. Hong , Y. Yuan , Z. Zhang , S. Lei , M. R. McCarter , A. Yadav , A. R. Damodaran , S. Das , G. A. Stone , J. Karapetrova , D. A. Walko , X. Zhang , L. W. Martin , R. Ramesh , L. Q. Chen , H. Wen , V. Gopalan , J. W. Freeland , Nat. Mater. 2019, 18, 377. 30886403 10.1038/s41563-019-0311-x

[97] S. K. Prasad , G. G. Nair , D. S. S. Rao , Liq. Cryst. 2009, 36, 705.

[98] K. A. Brekhov , K. A. Grishunin , D. V. Afanas'ev , S. V. Semin , N. E. Sherstyuk , G. K. Kitaeva , E. D. Mishina , T. Rasing , A. V. Kimel , JETP Lett. 2015, 102, 372.

[99] D. Lim , R. D. Averitt , J. Demsar , A. J. Taylor , N. Hur , S. W. Cheong , Appl. Phys. Lett. 2003, 83, 4800.

[100] P. Ruello , T. Pezeril , S. Avanesyan , G. Vaudel , V. Gusev , I. C. Infante , B. Dkhil , Appl. Phys. Lett. 2012, 100, 212906.

[101] M. Lejman , G. Vaudel , I. C. Infante , I. Chaban , T. Pezeril , M. Edely , G. F. Nataf , M. Guennou , J. Kreisel , V. E. Gusev , B. Dkhil , P. Ruello , Nat. Commun. 2016, 7, 12345. 27492493 10.1038/ncomms12345 PMC4980447

[102] C. Schmising , M. Bargheer , M. Kiel , N. Zhavoronkov , M. Woerner , T. Elsaesser , I. Vrejoiu , D. Hesse , M. Alexe , Phys. Rev. B 2006, 73, 212202. 10.1103/PhysRevLett.98.257601 17678054

[103] D. Schick , A. Bojahr , M. Herzog , P. Gaal , I. Vrejoiu , M. Bargheer , Phys. Rev. Lett. 2013, 110, 095502. 23496721 10.1103/PhysRevLett.110.095502

[104] Y. Li , C. Adamo , P. Chen , P. G. Evans , S. M. Nakhmanson , W. Parker , C. E. Rowland , R. D. Schaller , D. G. Schlom , D. A. Walko , H. Wen , Q. Zhang , Sci. Rep. 2015, 5, 16650. 26586421 10.1038/srep16650 PMC4653733

[105] J.‐G. Park , M. D. Le , J. Jeong , S. Lee , J. Phys.: Condens. Matter 2014, 26, 433202. 25299241 10.1088/0953-8984/26/43/433202

[106] P. Rovillain , R. de Sousa , Y. Gallais , A. Sacuto , M. A. Measson , D. Colson , A. Forget , M. Bibes , A. Barthelemy , M. Cazayous , Nat. Mater. 2010, 9, 975. 21076416 10.1038/nmat2899

[107] D. Talbayev , S. A. Trugman , S. Lee , H. T. Yi , S. W. Cheong , A. J. Taylor , Phys. Rev. B 2011, 83, 094403.

[108] E. Matsubara , T. Mochizuki , M. Nagai , M. Ashida , Phys. Rev. B 2016, 94, 054426.

[109] N. Ma , Y. Yang , Nano Energy 2018, 50, 417.

[110] B. Guzelturk , A. B. Mei , L. Zhang , L. Z. Tan , P. Donahue , A. G. Singh , D. G. Schlom , L. W. Martin , A. M. Lindenberg , Nano Lett. 2020, 20, 145. 31746607 10.1021/acs.nanolett.9b03484

[111] H.‐K. Nienhuys , V. Sundström , Phys. Rev. B 2005, 71, 235110.

[112] D. Issenmann , S. Schleef , S. Ibrahimkutty , G. Buth , T. Baumbach , A. Plech , M. Beyer , J. Demsar , Acta Phys. Pol. A 2012, 121, 319.

[113] M. Kozina , T. van Driel , M. Chollet , T. Sato , J. M. Glownia , S. Wandel , M. Radovic , U. Staub , M. C. Hoffmann , Struct. Dyn. 2017, 4, 054301. 28503632 10.1063/1.4983153 PMC5415405

[114] M. Misra , K. Kotani , I. Kawayama , H. Murakami , M. Tonouchi , Appl. Phys. Lett. 2005, 87, 182909.

[115] D. Daranciang , M. J. Highland , H. Wen , S. M. Young , N. C. Brandt , H. Y. Hwang , M. Vattilana , M. Nicoul , F. Quirin , J. Goodfellow , T. Qi , I. Grinberg , D. M. Fritz , M. Cammarata , D. Zhu , H. T. Lemke , D. A. Walko , E. M. Dufresne , Y. Li , J. Larsson , D. A. Reis , K. Sokolowski‐Tinten , K. A. Nelson , A. M. Rappe , P. H. Fuoss , G. B. Stephenson , A. M. Lindenberg , Phys. Rev. Lett. 2012, 108, 087601. 22463572 10.1103/PhysRevLett.108.087601

[116] J. Jahng , J. Brocious , D. A. Fishman , S. Yampolsky , D. Nowak , F. Huang , V. A. Apkarian , H. K. Wickramasinghe , E. O. Potma , Appl. Phys. Lett. 2015, 106, 083113.

[117] S. Yoshida , Y. Terada , M. Yokota , O. Takeuchi , H. Oigawa , H. Shigekawa , Eur. Phys. J.: Spec. Top. 2013, 222, 1161.

[118] S. Yoshida , Y. Aizawa , Z. H. Wang , R. Oshima , Y. Mera , E. Matsuyama , H. Oigawa , O. Takeuchi , H. Shigekawa , Nat. Nanotechnol. 2014, 9, 588. 24974938 10.1038/nnano.2014.125

[119] M. C. Fischer , J. W. Wilson , F. E. Robles , W. S. Warren , Rev. Sci. Instrum. 2016, 87, 031101. 27036751 10.1063/1.4943211 PMC4798998

